# Total protein, albumin and low-molecular-weight protein excretion in HIV-positive patients

**DOI:** 10.1186/1471-2369-13-85

**Published:** 2012-08-10

**Authors:** Lucy J Campbell, Tracy Dew, Rashim Salota, Emily Cheserem, Lisa Hamzah, Fowzia Ibrahim, Pantelis A Sarafidis, Caje F Moniz, Bruce M Hendry, Mary Poulton, Roy A Sherwood, Frank A Post

**Affiliations:** 1 Academic Department of Renal Sciences, King's College London, London, United Kingdom; 2 Departments of Clinical Biochemistry, King's College Hospital, London, United Kingdom; 3 Sexual Health, King's College Hospital, London, United Kingdom

**Keywords:** Proteinuria, Albuminuria, Retinol-binding protein, RBP, Cystatin C, Neutrophil gelatinase-associated lipocalin, NGAL, Tenofovir, HIV

## Abstract

**Background:**

Chronic kidney disease is common in HIV positive patients and renal tubular dysfunction has been reported in those receiving combination antiretroviral therapy (cART). Tenofovir (TFV) in particular has been linked to severe renal tubular disease as well as proximal tubular dysfunction. Markedly elevated urinary concentrations of retinal-binding protein (RBP) have been reported in patients with severe renal tubular disease, and low-molecular-weight proteins (LMWP) such as RBP may be useful in clinical practice to assess renal tubular function in patients receiving TFV. We analysed 3 LMWP as well as protein and albumin in the urine of a sample of HIV positive patients.

**Methods:**

In a cross-sectional fashion, total protein, albumin, RBP, cystatin C, and neutrophil gelatinase-associated lipocalin (NGAL) were quantified in random urine samples of 317 HIV positive outpatients and expressed as the ratio-to-creatinine (RBPCR, CCR and NGALCR). Exposure to cART was categorised as none, cART without TFV, and cART containing TFV and a non-nucleoside reverse-transcriptase-inhibitor (TFV/NNRTI) or TFV and a protease-inhibitor (TFV/PI).

**Results:**

Proteinuria was present in 10.4 % and microalbuminuria in 16.7 % of patients. Albumin accounted for approximately 10 % of total urinary protein. RBPCR was within the reference range in 95 % of patients while NGALCR was elevated in 67 % of patients. No overall differences in urine protein, albumin, and LMWP levels were observed among patients stratified by cART exposure, although a greater proportion of patients exposed to TFV/PI had RBPCR >38.8 μg/mmol (343 μg/g) (p = 0.003). In multivariate analyses, black ethnicity (OR 0.43, 95 % CI 0.24, 0.77) and eGFR <75 mL/min/1.73 m^2^ (OR 3.54, 95 % CI 1.61, 7.80) were independently associated with upper quartile (UQ) RBPCR. RBPCR correlated well to CCR (r^2^ = 0.71), but not to NGALCR, PCR or ACR.

**Conclusions:**

In HIV positive patients, proteinuria was predominantly of tubular origin and microalbuminuria was common. RBPCR in patients without overt renal tubular disease was generally within the reference range, including those receiving TFV. RBP therefore appears a promising biomarker for monitoring renal tubular function in patients receiving TFV and for distinguishing patients with normal tubular function or mild tubular dysfunction from those with severe renal tubular disease or Fanconi syndrome.

## Background

Chronic kidney disease (CKD) is present in approximately 15 % of HIV positive patients [1]. Uncontrolled HIV replication has been associated with the development of HIV-associated nephropathy (HIVAN) [2-4], CKD progression and loss of kidney function [3,5,6], while the use of combination antiretroviral therapy (cART) may improve renal function [7-9], reduce the incidence of acute renal failure [10], and delay progression to end-stage kidney disease [3,11,12]. However, specific antiretrovirals including tenofovir (TFV), indinavir and atazanavir have been associated with the development or progression of CKD [13-15], and current guidelines recommend screening for CKD at baseline in all HIV infected patients, and regularly thereafter for all or those at increased risk of CKD using estimated glomerular filtration rate (eGFR) and urinalysis [16-18].

Renal tubular disease and Fanconi syndrome have emerged as clinically significant complications of cART, and are most commonly observed with TFV [19]. The majority of reported cases of Fanconi syndrome have arisen in patients aged >40 years who received TFV together with didanosine or ritonavir-boosted protease inhibitors (TFV/PI) [20-23]. Milder forms of tubular dysfunction (defined by variable criteria) have been reported in 12-81 % of HIV positive patients on cART [24-27]. In these studies, tubular dysfunction was associated with older age [24,26-28], lower weight or BMI [26,27], diabetes mellitus [29], use of TFV [24,26,29-31] or TFV/PI [26], and with genetic polymorphisms in subjects exposed to TFV [28,32].

In patients with tubular dysfunction or Fanconi syndrome, an impaired ability of the proximal renal tubule to reabsorb phosphate, glucose, urate, amino acids and low molecular weight proteins (LMWP) from the glomerular ultrafiltrate results in increased urinary loss of these molecules. Retinol-binding protein (RBP) and cystatin C are examples of LMWP that are found in increased amounts in urine from patients with Fanconi syndrome [21,33]. It has been proposed that these biomarkers may be useful in the diagnosis of Fanconi syndrome and to monitor and detect tubular dysfunction in patients receiving TFV [34]. Neutrophil gelatinase-associated lipocalin (NGAL) is another LMWP, which is highly induced during inflammation and found to be a sensitive, early marker of acute kidney injury [35]. However, concentrations of these LMWP in urine of HIV positive patients and their associations with demographic and clinical parameters have not been well defined.

The objective of the present study was to examine the concentrations of different LMWP (RBP, cystatin C and NGAL) in relation to total protein and albumin excretion in urine of HIV positive patients, and to investigate possible factors associated with the highest quartile of urinary concentrations of these LMWP, with particular emphasis on the type of cART used.

## Methods

### Study population

We conducted a cross sectional study of consecutive HIV positive outpatients who attended King’s College Hospital, London, UK between 8/2006 and 8/2007. Patients were included if they agreed to participate in the study irrespective of the presence of kidney disease or risk factors for kidney disease. The study was approved by the local NHS research ethics committee (LREC) and all subjects provided informed consent.

For each subject, demographic, clinical and laboratory data were obtained from the clinic database and electronic hospital records. In addition, a detailed medical history was obtained, and height, weight, and waist circumference were measured. Three clinic blood pressure (BP) measurements were recorded of which the average of the second and third reading was used for the analysis. Blood samples were taken for routine biochemistry including renal, liver, bone, and lipid profiles, full blood count, CD4 T cell count and HIV RNA level. In addition, spot urine samples were obtained for measurement of creatinine, protein and albumin; an aliquot of urine was stored at −70°C for measurement of RBP, cystatin C and NGAL.

### Definitions

A diagnosis of hypertension was based on levels of systolic BP ≥140 or diastolic BP ≥90 mmHg or current treatment of hypertension with antihypertensive drugs [36]. Diabetes was defined by a prior diagnosis by a health care professional, use of anti-hyperglycaemic medication, or the need to follow a diabetic diet. eGFR was calculated with the 4-variable Modification of Diet in Renal Disease (MDRD) equation, incorporating age, gender and ethnicity [17]. Proteinuria was defined as urine protein-to-creatinine ratio (PCR) >20 mg/mmol (approximately >200 mg/g), macroalbuminuria as albumin-to-creatinine ratio (ACR) >30 mg/mmol (approximately >300 mg/g), and microalbuminuria as ACR 3–30 mg/mmol (approximately 30–300 mg/g), respectively. Exposure to cART was categorised as none, cART not containing TFV, cART containing TFV and a non-nucleoside reverse transcriptase inhibitor but no PI (TFV/NNRTI), or cART containing TFV/PI.

### Analytical methods

Urine albumin was measured using a polyethylene enhanced immunoturbidimetric assay, urine protein using the pyrogallol red method and creatinine with the kinetic Jaffe method on the ADVIA 2400 analyser (Siemens Healthcare Diagnostics Ltd, Camberley, UK). Retinol-binding protein was measured by enzyme-linked immunosorbent assay (ELISA; Immundiagnostik, Bensheim, Germany; reference range 0.01-0.54 mg/L), cystatin C by latex-enhanced immunoturbidimetric assay (Siemens Healthcare Diagnostics Ltd, Camberley, UK; no reference range supplied), and NGAL by ELISA (BioPorto Diagnostics, Gentofte, Denmark, reference range 0.7-9.8 μg/L), all with intra- and inter-assay variability of <5% and <10% respectively. For reasons of comparison, the urinary concentration of all LMWP was expressed relative to the urinary creatinine concentration (in mmol/L or g/L) as RBP/creatinine ratio (RBPCR), cystatin C/creatinine ratio (CCR), and NGAL/creatinine ratio (NGALCR).

### Statistical analysis

Statistical analyses were performed using STATA version 11 (StataCorp, College Station, TX, USA). Categorical variables are presented as absolute frequencies and the relevant percentages. Depending on the distribution, continuous variables are presented as means ± standard deviation (mean ± SD) or as medians with inter-quartile ranges. In view of their skewed distribution, PCR, ACR and LMWP measurements were also compared following log transformation. Clinical and laboratory parameters were described, and compared for patients stratified by cART regimen using chi-squared or Fisher’s exact test (categorical variables), Analysis of Variance or Kruskal-Wallis test (continuous variables). Correlations between the different LMWP and PCR were assessed using Pearson’s correlation coefficient, following log transformation of RBPCR and PCR, and square root transformation of CCR and NGALCR, in view of their skewed distribution.

The assays to quantify LMWP in urine are poorly standardised and the reference ranges for persons without kidney disease have not been widely validated. Hence, factors associated with the upper quartile (UQ) of RBPCR, CCR and NGALCR measurements were examined in logistic regression analyses. Variables were tested for interaction and included in the multivariate models if p was <0.05 in univariate analysis.

## Results

A total of 317 patients were studied. Demographic and clinical characteristics of the study cohort are depicted in Table 1. The mean age was 41years, 70% of patients were men, 60% of black ethnicity, and HIV infection was acquired through sex between men and IV drug use in 40% and 2% respectively. The median nadir and current CD4 count of the patients were 171 and 389 cells/mm^3^ respectively, 82% were taking cART at the time of sampling, and 65% had plasma HIV RNA levels <50 copies/mL. The median eGFR was 89 mL/min/1.73 m^2^; of note, only 4% and 2% of patients respectively had eGFR <60 and <50 mL/min/1.73 m^2^. Hypertension and diabetes mellitus was present in 15% and 4% of patients respectively, and 5% and 6% were co-infected with hepatitis B or C (Table 1).

As shown in Table 1, there were no significant differences between the groups of patients in terms of age, gender and ethnicity, with the exception of fewer men who received cART without tenofovir. Progressive increases from naive patients to patients receiving TFV/PI were noted in time since HIV diagnosis and time since starting cART. Patients receiving cART had lower nadir CD4 cell counts and more often experienced an AIDS-defining illness.

**Table 1:** Demographic and clinical characteristics of the total study population and the four study groups

		**All patients**	**No cART**	**cART/no TFV**	**cART/TFV**	**p-value**	**TFV/NNRTI**	**TFV/PI**	**p-value**
Number of patients	N (% of total)	317 (100)	57 (18)	143 (45)	117 (37)		72 (23)	45 (14)	
Age (years)	Mean (SD) (range)	41.0 (8.9) (24.4, 64.3)	38.0 (8.5) (21.2, 60.2)	40.9 (8.9) (25.8, 63.3)	42.3 (8.8) (25.7, 70.1)	0.83	42.3 (9.3) (21.7, 65.8)	42.6 (7.3) (27.5, 63.3)	0.30
Male gender	N (%)	222 (70)	43 (75)	**89 (62)**	90 (77)	0.02	54 (75)	36 (80)	0.53
Black Ethnicity	N (%)	189 (60)	31 (54)	95 (66)	63 (54)	0.08	35 (49)	28 (62)	0.15
Time since HIV diagnosis (years)	Median (IQR)	4.9 (2.1, 8.7)	**2.0 (0.5, 4.2)**	5.1 (2.2, 8.2)	5.8 (3.1, 4.7)	0.0001	5.1 (2.5, 8.2)	7.8 (4.1, 11.4)	0.003
Time since start cART (years)	Median (IQR)	4.6 (2.6, 7.6)	3.2 (2.6, 5.9)	4.3 (2.6, 6.8)	4.8 (2.6, 8.1)	0.51	4.5 (2.5, 7.3)	6.0 (3.1, 9.2)	0.009
AIDS (CDC status C)	(N, %)	77 (17)	**1 (2)**	27 (19)	33 (28)	<0.0001	17 (24)	16 (35)	0.16
Nadir CD4 count (cells/mm^3^)	Median (IQR)	171 (71, 262)	**290 (230, 416)**	161 (69, 250)	146 (64, 210)	0.0001	158 (82, 224)	94 (25, 222)	0.07
Current CD4 count (cells/mm^3^)	Median (IQR)	389 (270, 532)	437 (329, 560)	363 (269, 534)	385 (259, 517)	0.13	385 (245, 512)	376 (249, 566)	0.62
Current HIV RNA (log10)	Mean (SD)	2.4 (1.3)	**3.9 (1.1)**	2.2 (1.1)	2.0 (1.0)	0.0001	2.1 (1.1)	2.1 (1.0)	0.04
HIV RNA <50 c/mL	N (%)	204 (65)	**4 (9)**	110 (77)	90 (77)	<0.0001	60 (83)	30 (67)	<0.0001
Hepatitis B surface Ag (positive)	N (%)	15 (5)	3 (5)	4 (3)	8 (7)	0.31	4 (6)	4 (9)	0.49
Hepatitis C Antibody (positive)	N (%)	20 (6)	3 (5)	3 (2)	**14 (12)**	0.005	10 (14)	4 (9)	0.42
eGFR (mL/min/1.73 m2)	Median (IQR)	89 (78, 99)	91 (81, 99)	89 (80, 100)	85 (77, 97)	0.08	86 (76, 97)	82 (76, 96)	0.84
Diabetes	N (%)	11 (4)	0 (0)	7 (6)	4 (4)	0.27	2 (3)	2 (5)	0.62
Hypertension	N (%)	39 (15)	5 (12)	20 (16)	14 (13)	0.75	11 (17)	3 (8)	0.16
Weight (Kg)	Median (IQR)	74.2 (66.0, 82.0)	76.0 (68.9, 85.0)	74.3 (65.6, 82.0)	73.5 (66.0, 82.9)	0.39	73.6 (66.4, 81.0)	71.1 (65.6, 84.9)	0.73
BMI	Median (IQR)	24.6 (22.9, 28.1)	25.6 (22.5, 31.4)	25.5 (22.4, 29.6)	23.9 (21.9, 27.1)	0.06	24.1 (22.1, 26.8)	23.5 (21.4, 27.7)	0.73

p value for comparison between study groups (significance relates to the group in bold).

cART = combination antiretroviral therapy; TFV = tenofovir; NNRTI = non-nucleoside reverse transcriptase inhibitor; PI = protease inhibitor; eGFR = estimated glomerular filtration rate; BMI = body mass index.

**Figure 1:**
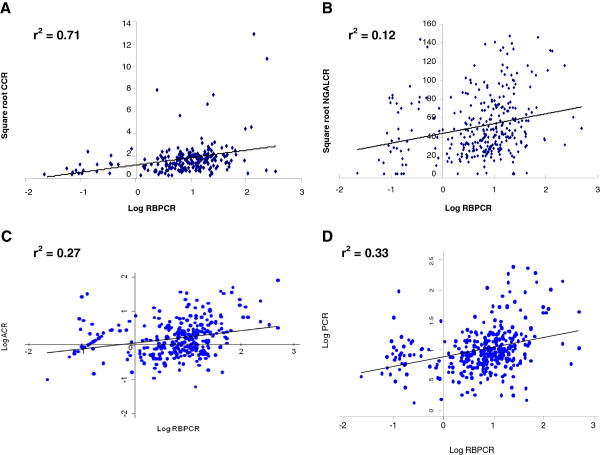
Correlations between RBPCR and CCR (A), NGALCR (B), ACR (C) and PCR (D).

Proteinuria, macroalbuminuria and microalbuminuria were present in 10.4%, 0.6% and 16.7% of patients respectively. There were no significant differences in the levels of urine protein and albumin excretion between the groups studied, although a trend towards lower values among patients not receiving cART was observed (Table 2). In patients with proteinuria, the median ratio of ACR/PCR was 9.9 (6.3, 27.0) percent, suggesting that albumin was a relatively small fraction of total urinary protein. Of the LMWP, good correlation between RBPCR and CCR was observed (r^2^ = 0.71 [95% CI 0.64, 0.77]) (Figure 1A). By contrast, NGALCR correlated poorly with RBPCR (r^2^ = 0.12 [0.008, 0.22]) (Figure 1B) or CCR (r^2^ = 0.03 [−0.09, 0.16]), and all LMWP correlated poorly with either PCR or ACR (r^2^ <0.4) (Figure 1C-D).

**Table 2:** Levels of total protein, albumin, low-molecular weight proteins in total and in the four study groups

		**All patients**	**No cART**	**cART/no TFV**	**cART/TFV**	**p-value**	**TFV/NNRTI**	**TFV/PI**	**p-value**
PCR (mg/g)	Median (IQR)	82.8 (56.2, 134.6)	73.4 (52.5, 111.8)	84.7 (58.8, 134.2)	82.7 (56.2, 141.9)	0.33	82.9 (60.9, 141.3)	82.6 (50.2, 158.0)	0.88
Log PCR (mg/g)	Mean (SD)	4.53 (0.85)	4.37 (0.66)	4.58 (0.93)	4.52 (0.82)	>0.05	4.53 (0.82)	4.50 (0.83)	0.82
ACR (mg/g)	Median (IQR)	10.3 (5.4, 22.2)	6.8 (4.9, 15.0)	11.5 (5.8, 28.9)	10.0 (5.7, 20.1)	0.10	10.5 (5.7 18.8)	9.6 (5.5, 24.9)	0.26
Log ACR (mg/g)	Mean (SD)	2.46 (1.16)	2.19 (1.07)	2.59 (1.26)	4.52 (0.82)	>0.05	2.36 (1.08)	2.48 (1.01)	0.24
RBPCR (μg/g)	Median (IQR)	66.4 (25.5, 150.4)	61.4 (15.1, 124.6)	60.4 (24.5, 130.4)	75.5 (28.2, 169)	0.24	75.23 (27.2, 138.7)	75.5 (30.6, 241.3)	0.27
Log RBPCR (μg/g)	Mean (SD)	3.92 (1.75)	3.64 (1.94)	3.86 (1.69)	4.13 (1.71)	>0.05	4.02 (1.66)	4.32 (1.78)	0.58
NGALCR (ng/g)	Median (IQR)	17993 (6998, 44823)	19658 (7919, 44728)	20049 (7172, 45319)	14412 (6011, 43756)	0.56	12770 (5491, 47963)	17993 (6998, 42732)	0.44
Log NGALCR (ng/g)	Mean (SD)	9.52 (1.99)	9.39 (2.36)	9.67 (1.82)	9.40 (1.99)	>0.05	9.40 (1.90)	9.40 (2.17)	0.99
CCR (μg/g)	Median (IQR)	2.44 (0.75, 5.39)	2.70 (0.79, 5.23)	2.82 (1.01, 5.51)	1.92 (0.56, 5.10)	0.13	1.92 (0.46, 5.02)	2.04 (0.70, 5.46)	0.44
Log CCR (μg/g)	Mean (SD)	0.80 (1.71)	0.64 (1.47)	1.12 (1.82)	0.50 (1.65)	0.02	0.35 (1.66)	0.76 (1.63)	0.29

p value for comparison between study groups.

PCR = protein-creatinine ratio (urine); ACR = albumin-creatinine ratio (urine); RBPCR = retinol-binding protein-creatinine ratio (urine); NGALCR = neutrophil gelatinase-associated lipocalin-creatinine ratio (urine); CCR = cystatin C-creatinine ratio (urine).

**Figure 2:**
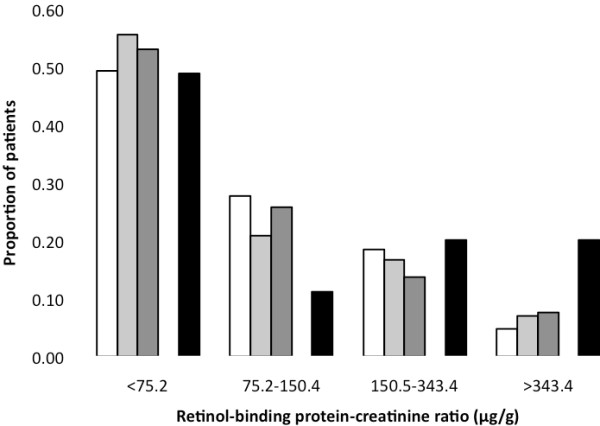
Proportion of patients from the four treatment groups at different levels of retinol-binding protein to creatinine ratio.* P = 0.003 (TFV/PI vs. other groups in patients with RBPCR >343.4 μg/g). Proportion of patients, stratified by cART exposure (no cART: open bars, cART without TFV: light grey bars, TFV/NNRTI: dark grey bars, TFV/PI: black bars) with retinol-binding protein-creatinine ratio values in the indicated range.

We observed no significant differences in LMWP excretion between the groups compared. However, RBPCR values >38.8 μg/mmol (>343.5 μg/g), a threshold previously associated with clinically significant renal tubular disease [21], was present in 5-8% of patients not receiving TFV/PI compared to 20 % of those receiving TFV/PI (p = 0.003), as shown in Figure 2. When applying the RBP and NGAL reference ranges provided by the manufacturers to our patients (using the median weight of 74.2 kg and assuming urine output of 0.5 mL/kg/hr), 17 (5.4%, including 4 patients exposed to TFV/NNRTI and 3 to TFV/PI) and 212 (66.9%) of our patients had RBPCR and NGALCR measurements above the upper limit (>48.1 μg/mmol [>425.8 μg/g] and >873 ng/mmol [>7724 ng/g], respectively).

In univariate analysis, older age, eGFR <90 mL/min/1.73 m^2^, longer time since HIV diagnosis, and treatment with TFV/PI signified a higher odds ratio of UQ RBPCR, whereas black ethnicity was associated with reduced odds of UQ RBPCR (Table 3). After adjustment, ethnicity and eGFR <75 mL/min/1.73 m^2^ were independently associated with UQ RBPCR, while a trend towards higher risk with TFV/PI was observed (p = 0.10). Longer duration of TFV exposure was not associated with higher RBPCR measurements (data not shown). Black ethnicity was the only factor associated with UQ NGALCR (OR 0.53 [0.32, 0.88], p = 0.01) and higher current CD4 cell counts the only factor associated with UQ CCR (OR 0.99 [0.99, 0.99], p = 0.03) in univariate analysis; multivariate analyses were thus not performed. Black ethnicity was marginally associated with UQ CCR (OR 0.59 [0.33, 1.08], p = 0.09) while impaired renal function and TFV/PI exposure were not related to higher odds of UQ NGAL or CCR.

**Table 3:** Crude and multivariate-adjusted odds ratios of upper quartile RBPCR

	**OR (95 % CI)**	**P-value**	**Adjusted OR (95 % CI)**	**P-value**
**Age (per 10 years increase)**	1.65 (1.24, 2.19)	<0.001	1.16 (0.82, 1.64)	0.46
**Gender (male v female)**	1.82 (0.99, 3.31)	0.05		
**Black ethnicity**	0.36 (0.22, 0.61)	<0.0001	**0.43 (0.24, 0.77)**	**0.005**
**Hypertension**	1.37 (0.65, 2.89)	0.40		
**Diabetes**	1.15 (0.29, 4.46)	0.84		
**HBsAg (positive)**	0.44 (0.09, 2.00)	0.29		
**HCV Antibody (positive)**	0.99 (0.04, 2.81)	0.98		
**eGFR**				
**≥90 mL/min/1.73 m2**	1		1	
**75-89 mL/min/1.73 m2**	1.91 (1.02, 3.57)	0.04	1.70 (0.88, 3.28)	0.13
**<75 mL/min/1.73 m2**	5.52 (2.78, 10.9)	<0.0001	**3.54 (1.61, 7.80)**	**0.001**
**Time since HIV diagnosis (per year increase)**	1.08 (1.03, 1.14)	0.003	1.02 (0.96, 1.08)	0.4
**Nadir CD4 count (per 50 cell increase)**	1.03 (0.95, 1.12)	0.47		
**Current CD4 count (per 50 cell increase)**	1.03 (0.98, 1.09)	0.28		
**Current HIV RNA <50 c/mL**	0.86 (0.51, 1.47)	0.61		
**AIDS (CDC status C)**	0.86 (0.04, 1.66)	0.65		
**cART status**				
**not on cART**	1		1	
**on cART not containing TFV**	1.13 (0.53, 2.37)	0.76	1.23 (0.51, 2.99)	0.65
**on TFV/NNRTI**	1.16 (0.50, 2.68)	0.73	1.06 (0.40, 2.78)	0.91
**on TFV/PI**	2.50 (1.04, 5.98)	0.04	2.40 (0.84, 6.86)	0.10

RBPCR = retinol-binding protein-creatinine ratio (urine);

## Discussion

This study aimed to evaluate urinary concentrations of three LMWP in relation to total urinary protein and albumin in a multiethnic HIV cohort and study potential associations of these parameters with the type of cART used. We observed poor correlations between LMWP and PCR or ACR, and no significant overall differences in urine PCR, ACR and LMWP excretion between patients stratified by cART and TFV exposure. RBPCR in 95% of patients was within the reference range. By contrast, 67% of patients had elevated NGALCR measurements.Black ethnicity was associated with reduced urinary concentrations of LMWP, and impaired renal function with increased urinary RBP concentrations.

Urinary RBP and cystatin C have previously been proposed as biomarkers for monitoring tubular function in HIV positive patients receiving TFV [25,33]. In a recent clinical trial, a 50% increase in urinary RBP excretion was observed in patients who initiated TFV with an NNRTI, with no change in the abacavir arm [30]. In a previous cross-sectional study, Hall and colleagues reported increased urinary RBP levels in patients receiving cART including TFV, compared to patients receiving cART without tenofovir or cART naive patients [25]. Our study differed from the latter study in that it included a 3-fold larger cohort, further stratified patients by TFV/NNRTI and TFV/PI exposure, and conducted a multivariate analysis of factors associated with UQ RBPCR measurements.

The absence of a significant association between raised RBPCR and TFV exposure in general, or TFV/PI exposure more specifically, may relate to the fact that our patients had no evidence of clinical renal tubular toxicity, the size and heterogeneity of the cohort, the assay or the RBPCR cut-off chosen for our analyses, or unmeasured confounding. Of note, the median RBPCR in a previous study of patients with TFV-induced Fanconi syndrome was 5593 μg/mmol (49,515 μg/g) [21], which is approximately 100-fold higher than the upper limit of the reference range of the assay used in our study. As almost all of our patients had RBPCR measurements within (or slightly above) the reference range, RBPCR appears to have good discriminatory value between severe, treatment-limiting renal tubular disease and normal tubular function or mild, asymptomatic renal tubular dysfunction.

HIVAN is the predominant cause of end-stage kidney disease in black patients with HIV infection [3,4,11,12]. In more advanced cases of HIVAN, urinary NGAL concentrations are typically elevated, and NGAL has been put forward as a useful urinary biomarker to aid the diagnosis of HIVAN [37,38]. Given the strong ethnic predisposition of HIVAN and that this condition may be present in patients with preserved renal function and mild proteinuria [39], it was surprising to find black ethnicity to be associated with lower odds of UQ NGALCR. However, as HIVAN affects only a small proportion of black patients in the UK [4], it is possible that increased urinary NGAL levels in those with (subclinical) HIVAN were masked by generally lower NGAL levels in black patients without HIVAN.

Our results suggest that RBPCR results may need to be interpreted in the context of eGFR and ethnicity. The observed association between ethnicity and tubular biomarkers is of interest and suggests that tubular handling of LMWP may be different in patients of different ethnic backgrounds, or that the prevalence of genetic polymorphisms of the organic anion transporters and/or multi-drug resistant proteins, which have been implicated in the pathogenesis of cART-associated tubular dysfunction [27,40], may vary by ethnicity. Alternatively, a higher muscle mass in black patients may have resulted in higher urinary creatinine concentrations, and thus somewhat lower RBPCR, NGALCR and CCR values.

In our cohort, NGALCR poorly correlated with either RBPCR or CCR. Although all these LMWP are generally proposed as biomarkers of proximal tubular dysfunction, the lack of correlation may suggest that urinary NGAL and RBP or cystatin C reflect different types and/or severity of tubular dysfunction. NGAL is considered to be a sensitive, early marker of acute kidney injury (acute tubular necrosis), i.e. of a state of extensive global injury of proximal tubular cells [35]. By contrast, RBP and cystatin C may be better markers of specific forms of proximal tubular dysfunction (such as inherited forms of Fanconi syndrome) [41] or states where proximal tubular dysfunction relates to mitochondrial toxicity – and thus a decrease in energy supply - and not to actual necrosis [21].

In the FRAM study, microalbuminuria was present in 11% of HIV positive patients, and associated with several cardiovascular risk factors including insulin resistance and hypertension, as well as immunodeficiency [42]. Several studies in the general population suggest that urine albumin excretion at the level of microalbuminuria associates with increased risk of cardiovascular events and death, whereas protein excretion at the level of macroalbuminuria or clinical proteinuria (i.e. urine protein >0.5 g/day) predicts both cardiovascular events and CKD progression [43]. The associations of elevated albumin and protein excretion with future risks of developing renal failure, cardiovascular events and death have also been confirmed in studies in HIV positive patients [44-47], and thus, urine albumin and protein excretion should be assessed and taken into account in cardiovascular and CKD risk reduction strategies.

This study has several limitations. It followed a cross-sectional design and, thus, longitudinal associations could not be evaluated. We relied on a single PCR, ACR and LMWP measurement for our analyses, and we lacked data on phosphate reabsorption or renal histology. Despite the overall sample size, the number of patients receiving TFV/PI was relatively small, and HIV negative controls were not included. Nonetheless, the ethnically diverse participants in our study are representative of the HIV population in the UK, and patients were only selected by their willingness to participate in the study and not for the presence or absence of specific clinical characteristics.

## Conclusions

This study suggests that urinary LMWP concentrations in HIV positive patients are unrelated to viral replication or exposure to cART in general, or exposure to TFV specifically. RBPCR was within the reported range in 95% of our study subjects, suggesting that RBP may be a useful marker to distinguish patients with normal tubular function or mild tubular dysfunction from those with clinically significant renal tubular disease and Fanconi syndrome.

## Competing interests

EC, LH and FAP have received funding to attend conferences or educational meetings from GlaxoSmithKline, ViiV healthcare, Bristol-Myers Squibb, Jansen-Cilag, Abbott Laboratories and Gilead Sciences. BMH has received honoraria for consultancy from Abbott Laboratories, AM Pharma and Gilead Sciences. RAS has received honoraria for presentations at conferences and preparation of educational material from Siemens Healthcare Diagnostics. FAP has received honoraria from Gilead Sciences, Bristol-Myers Squibb, Janssen-Cilag, GlaxoSmithKline, ViiV healthcare, Merck and Roche, and research funding from Bristol-Myers Squibb, GlaxoSmithKline and ViiV healthcare. All others: no conflict.

## Authors’ contributions

CFM, BMH, RAS and FAP designed the study. LJC, EC, LH and FAP collected samples and clinical information. TD, RS, RAS and CFM performed the laboratory assays. LJC performed the statistical analyses with input from FI, CFM, BMH, MP, RAS and FAP. FAP and PAS wrote the manuscript with input from all authors. The final version of the manuscript was approved by all authors.

## Pre-publication history

The pre-publication history for this paper can be accessed here:

http://www.biomedcentral.com/1471-2369/13/85/prepub
